# SUMOylation of HP1α supports association with ncRNA to define responsiveness of breast cancer cells to chemotherapy

**DOI:** 10.18632/oncotarget.8733

**Published:** 2016-04-14

**Authors:** Feng-Ming Lin, Santosh Kumar, Jing Ren, Samaneh Karami, Shaymaa Bahnassy, Yue Li, Xiaofeng Zheng, Jing Wang, Tasneem Bawa-Khalfe

**Affiliations:** ^1^ Department of Cardiology, The University of Texas MD Anderson Cancer Center, Houston, TX, USA; ^2^ Center for Nuclear Receptors and Cell Signaling, Department of Biology and Biochemistry, University of Houston, Houston, TX, USA; ^3^ Department of Integrative Biology and Pharmacology, University of Texas Health Science Center at Houston, Houston, TX, USA; ^4^ Department of Bioinformatics and Computational Biology, The University of Texas MD Anderson Cancer Center, Houston, TX, USA

**Keywords:** SUMO, SENP7, HP1α, ncRNA, Rad51C

## Abstract

Epigenetic reprogramming allows cancer cells to bypass normal checkpoints and potentiate aberrant proliferation. Several chromatin regulators are subject to reversible SUMO-modification but little is known about how SUMOylation of chromatin-remodelers modulates the cancer epigenome. Recently, we demonstrated that SUMO-protease SENP7L is upregulated in aggressive BCa and maintains hypoSUMOylated heterochromatin protein 1-α (HP1α). Canonical models define HP1α as a “reader” of repressive H3K9m3 marks that supports constitutive heterochromatin. It is unclear how SUMOylation affects HP1α function in BCa cells. This report shows HP1α SUMO-dynamics are closely regulated in a complex with SENP7L and SUMO-E3 Polycomb-2 (PC2/CBX4). This complex accumulates at H3K9m3 sites, hypoSUMOylates HP1α and PC2, and reduces PC2's SUMO-E3 activity. HyperSUMO conditions cause complex dissociation, SUMOylation of PC2 and HP1α, and recruitment of SUMOylated HP1α to multiple DNA-repair genes including *Rad51C*. SUMOylated HP1α's enrichment at euchromatin requires chromatin-bound non-coding RNA (ncRNA), reduces Rad51C protein, and increases DNA-breaks in BCa cells. Hence, HP1α SUMOylation and consistently low SENP7L increase efficacy of DNA-damaging chemotherapeutic agents. BCa patients on chemotherapy that express low SENP7L exhibit greater survival rates than patients with high SENP7L. Collectively, these studies suggest that SUMOylated HP1α is a critical epigenetic-regulator of DNA-repair in BCa that could define chemotherapy responsiveness.

## INTRODUCTION

SUMO post-translational modification (PTM) of protein substrates is important for normal cell physiology but is thrown off kilter with the onset of cancer [1, 2]. This process or SUMOylation requires one or more of 3 SUMO isoforms, E1 activating molecules, and a sole E2 conjugating enzyme Ubc9. Often SUMO E3 ligases facilitate interaction between Ubc9 and the target protein and thereby potentiate the conjugation reaction. In contrast, SENPs reverse this PTM or deSUMOylate *via* hydrolysis of the isopeptide bond between SUMO and the target [3, 4]. The two arms of SUMO-PTM work in concert to dictate the level of SUMOylated cellular substrates.

Several components of the SUMO machinery are localized at select chromatin loci. We and others have demonstrated that full-length SUMO protease SENP7L is present at the tri-methylated histone 3 lysine 9 residue (H3K9m3, [5–7]). SUMO E3 ligase Polycomb-2 (PC2/CBX4) can associate with H3K9m3 and an additional repressive mark tri-methylated histone 3 lysine 27 residue (H3K27m3) as established in recombinant *in vitro* systems [8]. *In vivo* studies indicate that recruitment of PC2 to H3K27m3 is potentiated with SUMO-PTM of PC2 [9]; in contrast, little is known about what regulates localization of PC2 to H3K9m3. Enrichment of SENP7L to H3K9m3 requires interaction with the canonical H3K9m3 reader heterochromatin protein-1 alpha (HP1α).

Previous studies established the critical role of HP1α in stabilizing constitutive heterochromatic regions, specifically the pericentromere and subtelomere [10, 11]. Recent studies provide increasing evidence to support the recruitment and function of HP1α at transcriptional active euchromatin sites [5, 12, 13]. Unlike HP1α's recruitment to heterochromatin, little is known about what directs HP1α to euchromatin sites. While heterochromatin is H3K9m3-rich, HP1α is present at the pericentromeric region prior to the establishment of H3K9m3. H3K9m3-independent recruitment of HP1α to the pericentromere occurs predominantly *via* HP1α's association with *cis*-acting non-coding RNA (ncRNA) of the α-satellite repeats [7, 10, 14]. It is unknown whether chromatin-bound ncRNA could direct HP1α to euchromatin sites.

Recently we reported that HP1α's enrichment to select euchromatin loci dictates the aggressiveness of breast cancer (BCa) cells [5]. In BCa cells, SUMO-PTM of HP1α facilitates HP1α's localization to the promoter of proliferation- and mesenchymal-inducing genes and subsequently silences transcription of these genes. Immunofluorescence data shows persistent binding of SUMOylated HP1α outside constitutive heterochromatin. However, chromatin-binding profiles of HP1α are lacking. This would provide greater understanding of HP1α's function in BCa especially as we and others have demonstrated HP1α levels correlate with metastatic disease [5, 15–17]. In highly invasive BCa cells, HP1α is hypoSUMOylated but it is unclear whether reduction of HP1α SUMOylation affects transcription of additional BCa cell survival genes.

In the present study, we define how and why SUMOylation of HP1α is closely guarded in BCa cells. HP1α concurrently binds the SUMO E3 ligase PC2 and isopeptidase SENP7L; this complex maintains hypoSUMOylated HP1α levels in BCa cells. Knockdown of SENP7L or PC2 overexpression increases HP1α SUMOylation. HyperSUMOylated HP1α exhibits an altered chromatin-binding profile as compared to its un-modified counterpart with elevated binding to 5′-UTR and promoter regions of multiple genes including DNA-damage response (DDR) genes. Recruitment of the SUMOylated HP1α to these euchromatic sites requires chromatin-bound ncRNA in BCa cells. HP1α hyperSUMOyation and SENP7L depletion increase the sensitivity of BCa cells to DNA-damaging chemotherapeutics. Consistently BCa patients with lower SENP7L exhibit a better response to chemotherapy.

## RESULTS

### PC2 serves as SUMO E3 ligase and interacting partner for HP1α

**Figure 1:**
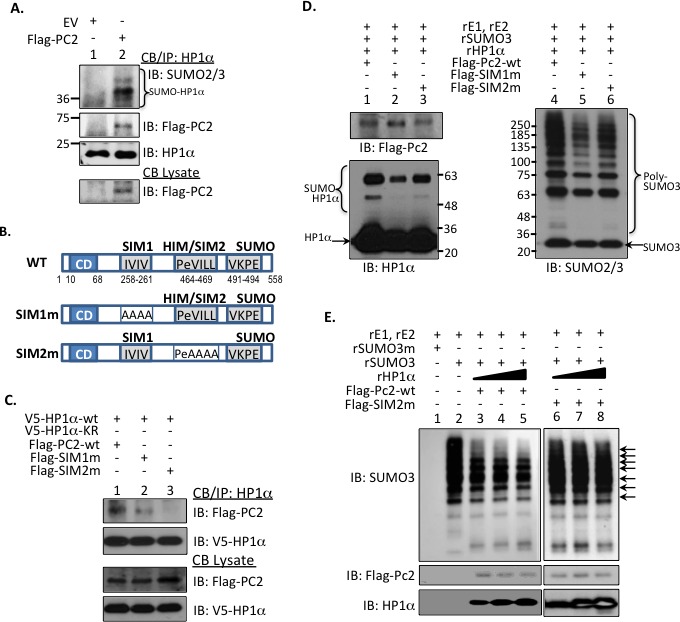
HP1α interacts with PC2 to regulate PC's SUMO E3 ligase activity **A.** Chromatin-bound endogenous HP1α associates with and is a target for PC2. Endogenous HP1α was immunoprecipitated from MCF7 cells expressing empty vector (EV) or flag-tagged PC2. Immunoblots using SUMO2/3 and Flag antibodies confirmed HP1α modification and protein-protein interaction. **B.** Schematic representation of PC2's 2 SUMO-interaction motifs (SIM), HP1α-interaction motif (HIM), and single SUMO-conjugation site in relation to the chromodomain (CD). The hydrophobic amino acids were mutated to alanine to generate the appropriate SIM1 and SIM2 mutants. **C.** HP1α-interaction motif is required for PC2-HP1α binding. MCF7 cells were incubated with either wild-type or PC2 plasmids with mutations to either 1 of the 2 SIMs. Association of PC2 with HP1α was assessed *via* SDS-PAGE. **D.** PC2-SIM1 affects *in vitro* HP1α SUMOylation. Purified Flag-tagged wild-type or SIM-mutant PC2 was incubated with recombinant SUMO machinery and HP1α and subsequently subject to immunoblotting. Poly-SUMO chains (brackets labeling SUMO-HP1α and Poly-SUMO3) and unmodified HP1α and SUMO3 protein (arrows) is indicated. **E.** The E3 activity of wt-PC2, but not SIM2m, is reduced with increasing concentrations of HP1α. An *in vitro* SUMOylation assay was used to evaluate poly-SUMO chains in the presence of increasing recombinant HP1α. Arrows SUMO3 bands in PC2-wt samples affected by the addition of recombinant HP1α (rHP1α).

Recently, we reported that HP1α is more readily SUMOylated in non-cancerous *versus* cancer mammary epithelial cells [5]. However, it is unknown what components of the SUMO-conjugating machinery facilitate HP1α SUMOylation. Since PC2 also interacts with H3K9m3 sites, we postulated that overexpression of this SUMO E3 ligase would potentiate SUMO-PTM of HP1α. Indeed, isolated endogenous chromatin-bound HP1α is readily SUMOylated with induction of PC2 in MCF7 cells (bracket highlights SUMOylated HP1α, Figure 1A). While PC2 substantially enhances HP1α SUMOylation, co-transfection of HP1α with other chromatin-associated SUMO E3 ligases, specifically protein inhibitor of activated STAT 4 (PIAS4), LIM domain only 2 (LMO2), and RAN binding protein 2 (RanBP2, [9, 18–20]) only modestly increases poly-SUMO modification of HP1α (Figure S1A).

Interestingly, PC2 directly interacts with its substrate HP1α in chromatin fractions (Figure 1A). The amino acid sequence of PC2 includes two well-defined SUMO-interacting motifs (SIM) as well as a lysine residue near the C-terminus that serves as a SUMO-acceptor site [21, 22]. Previous studies established that PC2 requires both SIMs for SUMO E3 ligase activity and PC2 auto-SUMOylation [21, 22]. PC2 also includes a potential HP1α-interaction motif (HIM) or PxVxL sequence that exists as part of the second SIM (SIM2, Figure 1B). Hence we evaluated how critical the converging amino acids of the HIM/SIM2 sequence are for PC2's interaction with HP1α. Wild-type forms of HP1α and PC2 bind efficiently (lane 1, Figure 1C). While mutation of IVIV-SIM1 sequence (SIM1m) reduces interaction with HP1α-wt (lane 2, Figure 1C), replacement of the VILL sequence (SIM2m) ablates the ability of PC2 to bind HP1α-wt (lane 3, Figure 1C). Hence, SIM2 overlaps with a canonical HIM, which supports PC2 binding to HP1α.

Compared to SIM2m, SIM1m promotes less *in vitro* SUMOylation of HP1α (lane 2 *versus* lane 3, Figure 1D) and SUMO3 poly-chain formation (lane 5 *versus* lane 6, Figure 1D). This suggests SIM1, more than SIM2, contributes to PC2's E3 ligase function. However, both PC2-SIMs are required for optimum enzymatic activity (lane 1 and lane 4, Figure 1D). We next investigated whether association with HP1α affects PC2's ligase function. In a cell-free system, increasing amounts of recombinant HP1α protein reduce the ability of wt-PC2 to initiate poly-SUMO3 chain formation (lane 3-5, Figure 1E). Partial loss of HIM (SIM2m) restores SUMO E3 activity even with the addition of increasing recombinant HP1α (lane 6-8, Figure 1E). Inversely, inhibition of SUMO ligase activity regulates PC2's interaction with HP1α. PC2 expresses a serine-SIM consensus sequence [21]; on an additional SUMO ligase PIAS1, casein kinase 2 (CK2) phosphorylates the serine residue adjacent to a SIM to potentiate ligase activity [23]. Treatment with a CK2 inhibitor reduces PC2 phosphorylation (Supplemental Figure S1B) and SUMOylation (Supplemental Figure S1C). This reduction of PC2 auto-SUMOylation and corresponding E3-activity concurrently increases PC2-HP1α interaction (Supplemental Figure S1C).

### HP1α interacts with SUMO E3-ligase PC2 and Isopeptidase SENP7L simultaneously

**Figure 2:**
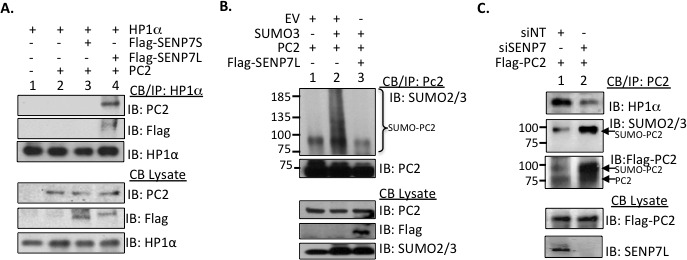
SENP7L regulates PC2-HP1α interaction and PC2 SUMOylation **A.** PC2, HP1α, and SENP7L interact at the chromatin. Chromatin-bound HP1α was isolated from MCF7 cells and interaction with PC2 and/or select SENP7 isoforms was evaluated with Western Blot analysis. **B.** SENP7L deSUMOylates PC2. In *in vivo* SUMOylation studies, modification of isolated chromatin-bound PC2 is identified with SUMO2/3 antibody in the presence and absence of SENP7L. **C.** SENP7L dictates PC2's SUMOylation and binding to HP1α. MCF7 cells were treated with the appropriate siRNA for 48hr and subsequently the modification state of the chromatin-bound PC2 was established as described above.

HP1α interacts with SENP7L through multiple HIMs [5–7]. Hence we questioned whether HP1α associates with PC2 and SENP7L simultaneously or independently. Chromatin-bound HP1α efficiently interacts with PC2 following induction of SENP7L in MCF7 cells (lane 4, Figure 2A). A SENP7 isoform, which lacks one HIM sequence (SENP7S), neither binds HP1α (lane 3, Figure 2A and [5]) nor supports PC2-HP1α interaction (lane 3, Figure 2A). While increasing SUMO3 supports poly-SUMO conjugation of PC2, concurrent SENP7L induction prompts deSUMOylation of chromatin-bound PC2 (lane 2 and 3, respectively, Figure 2B). Inversely, siRNA-targeted knockdown of SENP7 (siSENP7) enhances PC2 SUMOylation in MCF7 cells (Figure 2C). The same SENP7 knockdown conditions also increase HP1α SUMOylation and concomitant PC2 overexpression further potentiates the SUMOylated HP1α population (Supplemental Figure S2A and S2B). Hence, both PC2 and HP1α are substrates for the isopeptidase SENP7L. Interestingly, the loss of SENP7 also reduces PC2's interaction with HP1α (Figure 2C), suggesting that SENP7-mediated deSUMOylation is important for PC2-HP1α binding.

**Figure 3:**
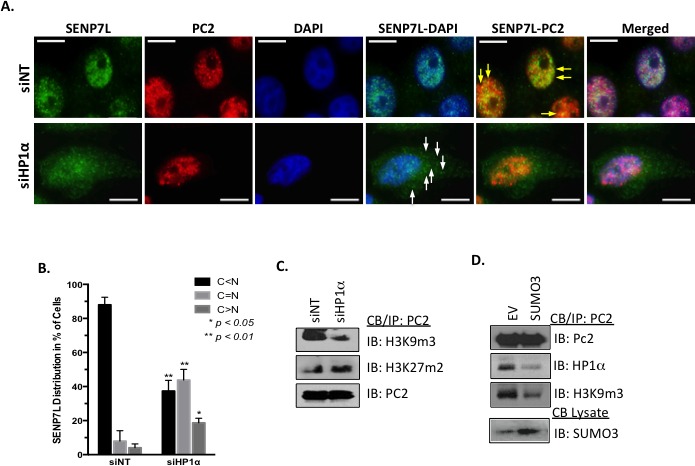
HP1α directs the localization of PC2 and SENP7L **A.**-**B.** HP1α loss alters SENP7L subcellular localization. Cells were incubated with either non-targeting (siNT) or HP1α-specific (siHP1α) siRNA for 48 hr. Immunofluorescence was performed with SENP7L and PC2 antibodies and nuclei stained with DAPI. Total cells and SENP7L distribution was evaluated in randomly selected 20x magnification fields (siNT: *n* = 3 and siHP1α: *n* = 7); percent of cells with cytosolic (**C)** *versus* nuclear (N) SENP7L were analyzed using ANOVA and Tukey's post-hoc. **C.** PC2's enrichment at H3K9m3 requires HP1α. Chromatin-bound PC2 was isolated from MCF7 cells treated with either siNT or siHP1 as described above. Western blot analysis was performed to evaluate PC2's association with modified histones tails, specifically H3K9m3 and H3K27m3. **D.** HyperSUMO conditions reduce PC2's association with both H3K9m3 and HP1α. MCF7 cells were exposed to elevated SUMO3 levels for 24 hr and binding partners of endogenous PC2 was evaluated using the immunoprecipitation/ immunoblot techniques.

Since both PC2 (Figure 1C) and SENP7L express HIMs, it is likely that HP1α serves as a scaffold for the recruitment of both SUMO components to the H3K9m3 mark. Both PC2 and SENP7L localize to the nucleus predominantly (yellow arrows, Figure 3A). Treatment of MCF7 cells with siRNA that efficiently reduces HP1α protein levels (siHP1α, Supplemental Figure S3A) alters the subcellular distribution of SENP7L but not PC2. Specifically, HP1α loss increases SENP7L localization to the cytosol (white arrows, Figure 3A); BCa cells expressing SENP7L in the nucleus are reduced while cells with even or greater distribution of SENP7L in the cytosol are significantly increased (*p < 0.01*, Student's *t*-test, Figure 3B and Supplemental Figure S3B). This observation correlates with previous studies that HP1α is required for localization of SENP7 in the nucleus and more specifically at the constitutive pericentric heterochromatin [7, 24]. Although siHP1α treatment did not alter subcellular distribution of PC2 (Figure 3A), HP1α loss decreases the enrichment of PC2 at H3K9m3 marks (Figure 3C). Inversely, PC2's localization at H3K27m3 sites (Figure 3C) and SUMOylation (Supplemental Figure S3C) is enhanced with HP1α knockdown. Similarly, hyperSUMO conditions reduce PC2's recruitment to H3K9m3 sites (Figure 3D). PC2-HP1α interaction also diminishes with SUMO3 induction (Figure 3C); analogously PC2-HP1α binding decreases with enhanced SUMOylation of PC2 (Figure 2C). Hence, it is likely that un-modified PC2 associates predominantly with HP1α at the H3K9m3 mark.

### SUMOylated HP1α is recruited to ncRNA-richchromatin loci

**Figure 4:**
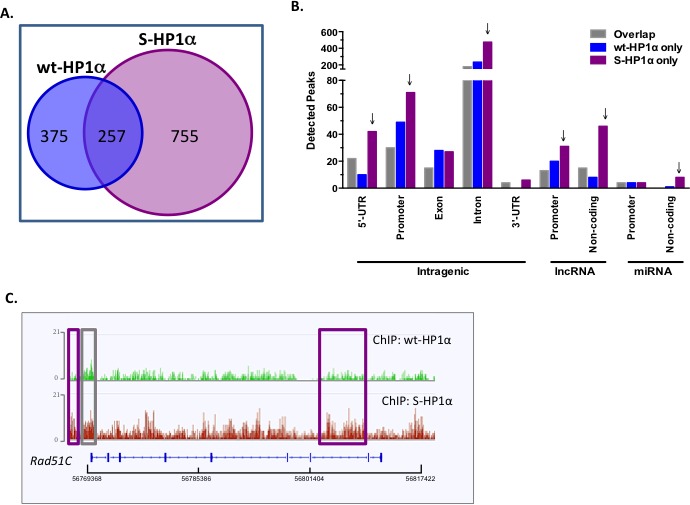
HP1α SUMOylation alters its chromatin binding profile **A.** Venn diagram of the total binding loci of wild-type and SUMO3-fused HP1α (wt- and S-HP1α) as identified by ChIP-Seq analysis. **B.** S-HP1α binds more 5′-UTR, promoters, and introns in intragenic regions and regions transcribing ncRNA. Graph represents distribution of detected peaks (binding sites) of wt and S-HP1α at indicated intragenic region. Gray bars indicate binding sites shared by both HP1α types (Overlap) while binding sites unique to wt-HP1α or S-HP1α only are labeled appropriately. Arrows highlight chromosomal regions with a difference between binding profiles of S-HP1α and wt-HP1α. **C.** ChIP-Seq track at the *Rad51C* loci that highlights the number and position of the binding sites for wt- and S-HP1α. Purple box indicates a unique S-HP1α binding site while gray box is common to both wt and S-HP1α.

A previous report suggests that interaction with mouse SENP7 docks HP1α at H3K9m3-rich pericentromeric heterochromatin [24]. Interestingly, hyperSUMOylated HP1α does not efficiently bind either SENP7 or PC2 (Supplemental Figure S3D). Hence, we postulated that SUMOylated HP1α could exhibit a different chromatin binding profile than its unmodified counterpart. To test this, ChIP-Seq experiments were utilized to evaluate the distribution of wild-type HP1α (wt-HP1α) *versus* a SUMOylated HP1α mimetic (S-HP1) that includes HA-tagged SUMO3 close to the endogenous SUMO-acceptor site of HP1α [5]. A high number of sequencing reads from the samples aligned to the hg19 reference genome (Supplemental Table S1). Biostatistical validation was performed to filter peaks based on high confidence (FDR 0.05) and no overlap with input peaks (Supplemental Table S2). Of the 632 and 1012 filtered peaks for wt-HP1 and S-HP1α respectively, the two constructs share only 257 binding sites (Figure 4A). The S-HP1α has a greater number of “unique” binding sites or regions that do not overlap with wt-HP1α (Figure 4A). These unique S-HP1α binding sites are detected at gene-coding and non-coding loci; specifically, S-HP1 binds the 5′UTRs, promoters, and introns in intragenic regions as well as promoters of long ncRNA (lncRNA) and non-coding regions for both lncRNA and microRNA (miRNA) (Figure 4B). Many of the novel loci for S-HP1α include genes that contribute to the DDR, specifically *Rad51C, PPM1D, PPM1E, BRIP1*. Alignment of the binding profiles of wt- *versus* S-HP1α on the *Rad51C* gene loci exhibit interesting similarities and differences (Figure 4C). Both wt- and S-HP1 bind at the *Rad51C* promoter and 5′-UTR (gray box, Figure 4C) but a greater detection of S-HP1α is observed adjacent to the promoter and in intron 7 (purple box, Figure 4C).

**Figure 5:**
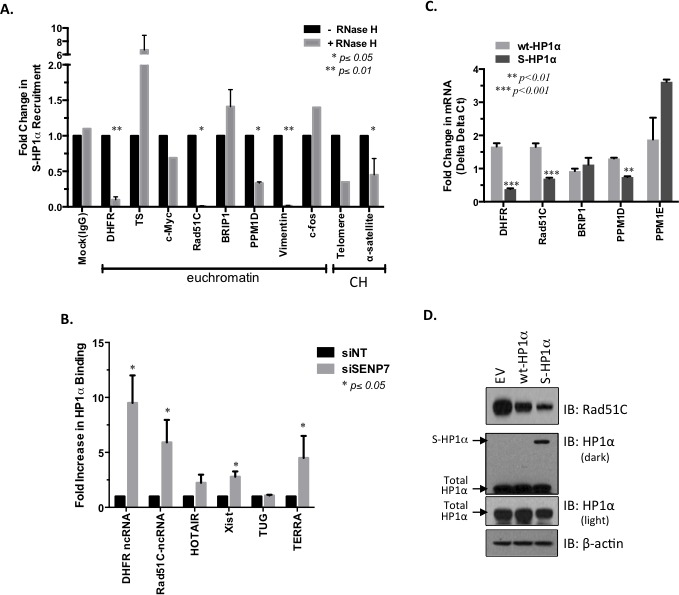
ncRNA-mediated recruitment of SUMOylated HP1α to DDR-regulating gene loci **A.** RNA/DNA complex required for the recruitment of HP1α to select chromatin loci. MCF7 cells were treated with RNase H to disrupt RNA-DNA interaction prior to ChIP of the S-HP1α protein. Real-time PCR was performed to identify S-HP1α recruitment to specific chromatin loci; CH represents constitutive heterochromatin sites. Student's *t*-test on raw C_T_ data was used to evaluate for statistical significance with *p*-values < 0.05 indicated with an asterisk. **B.** HP1α associates with lncRNA in the absence of SENP7L. Following either non-targeting or SENP7-specific siRNA treatment for 48 hr, chromatin-bound RNA that associates with HP1α was isolated *via* chromatin-RNA immunoprecipitation assay (Ch-RIP). Primers for select ncRNA were used to detect the immunoprecipitated RNA from each treatment group. Bar graph indicates the mean ± SEM of three independent Ch-RIP experiments. **C.** SUMOylated HP1α reduces select DDR gene transcripts. An empty vector, wild-type HP1α (wt-HP1α), or SUMO3-fused HP1α (S-HP1α) plasmid was overexpressed in MCF7 cells and RNA was isolated. Subsequently real-time PCR analysis was conducted with the appropriate primers. The graph represents mRNA levels of either wt-HP1α or S-HP1α treated cells normalized to levels in cells with empty vector; Student's *t*-test indicated significance. **D.** SUMOylated HP1α reduces Rad51C protein. Cells expressing either empty vector (EV), wild-type HP1α, or S-HP1α were lysed and subject to SDS-PAGE to evaluate Rad51C levels. Total HP1α represents both endogenous and exogenous HP1α. Blots are representative of 2 independent experiments.

S-HP1α appears readily bound to genes like *Rad51C* that express unspliced lncRNA (Figure 4B). A previous report suggests that SUMOylated HP1α interacts more readily with ncRNA at the constitutive heterochromatin in mouse fibroblasts [14]. Hence we first evaluated the binding profile of wt- and S-HP1α with published *trans*-binding regions for 2 lncRNAs TERC and HOTAIR [25]. Significantly more S-HP1α (33 sites), but not wt-HP1α (4 sites), binding sites either directly overlapped with or were within close proximity (200bp) to either TERC or HOTAIR *trans-*binding regions. To further test whether ncRNA facilitate SUMOylated HP1α‘s recruitment to active chromatin sites, a modified ChIP protocol was developed. The RNA-DNA complex was disrupted with RNase H treatment prior to ChIP with the HP1α antibody; the treatment did not cause adverse RNA and/or DNA degradation in the harvested cell fractions. The recruitment of S-HP1α to the pericentromeric α-satellite repeats was significantly attenuated with RNase H treatment (Figure 5A). This is consistent with previous immunofluorescence studies in which hyperSUMO conditions promote the α-satellite-dependent recruitment of HP1α to the pericentromere [14]. In addition, the enrichment of S-HP1α at the heterochromatic telomere is reduced with RNase H treatment (Figure 5A). An intact RNA-DNA complex is also required for the localization of S-HP1α to several, but not all, ChIP-Seq-identified euchromatic sites (Figure 5A). Specifically RNase treatment only affects S- HP1α enrichment at the E2F-responsive gene *DHFR* but not *TS* or *c-Myc* (*p < 0.01*, Student's *t*-test, Figure 5A). Similarly, loss of the RNA-DNA complex affects S-HP1α at the *Rad51C* and *PPM1D* gene loci but not *BRIP1* (*p < 0.05*, Student's *t*-test, Figure 5A). Interestingly, S-HP1α is lost at chromatin loci that exhibit known and predicted ncRNA; specifically *DHFR, Rad51C, PPM1D, Vimentin, telomere,* and *α-satellite* (Figure 5A, [26–28]).

To evaluate the relationship between HP1α and ncRNA further, chromatin-associated RNA was isolated with a chromatin-RIP (Ch-RIP). Prior to Ch-RIP, hyperSUMOylation of HP1α was achieved in MCF7 cells with siRNA-targeted knockdown of HP1α -interacting partner SENP7L (Supplemental Figure S2A and previously [5]. As others have established the ability of HP1α to interact with ncRNA at the pericentromeric and subtelomeric region [14, 29, 30], HP1α's association with the telomeric TERRA ncRNA served as control. HyperSUMOylation of HP1α, with SENP7L reduction, enhances the ability of HP1α to bind *cis*-acting ncRNA TERRA and Xist (Figure 5B). A more modest, but not statistically significant, interaction between HP1α and *trans*-acting HOTAIR is observed while no change in TUG ncRNA is detected with SENP7 knockdown (Figure 5B). SENP7-siRNA treatment significantly increases HP1α's association with two additional ncRNA, promoter-associated DHFR-ncRNA and the ncRNA transcript of the *Rad51C* gene NR_103873 (Figure 5B). While the DHFR-ncRNA is known to mediate *DHFR* transcription [26, 27], the function of Rad51C-ncRNA is unknown. Our data suggests that the Rad51C ncRNA can be isolated in chromatin-bound RNA fractions and interacts with HP1α (Figure 5B).

The recruitment of HP1α to the *Rad51C* promoter serves to regulate expression of Rad51C mRNA (Figure 5C and primers Supplemental Table S5). Induction of wt-HP1α consistently also reduces Rad51C protein levels as compared to empty vector control (lane 2 *versus* lane 1, Figure 5D). SUMO PTM of HP1α potentiates this response as the most dramatic loss of Rad51C is observed with overexpression of S-HP1α in MCF7 cells (lane 3 *versus* lanes 1 and 2, respectively, Figure 5D).

### HP1α SUMOylation promotes DNA-damage and correlates with chemotherapy sensitivity

**Figure 6:**
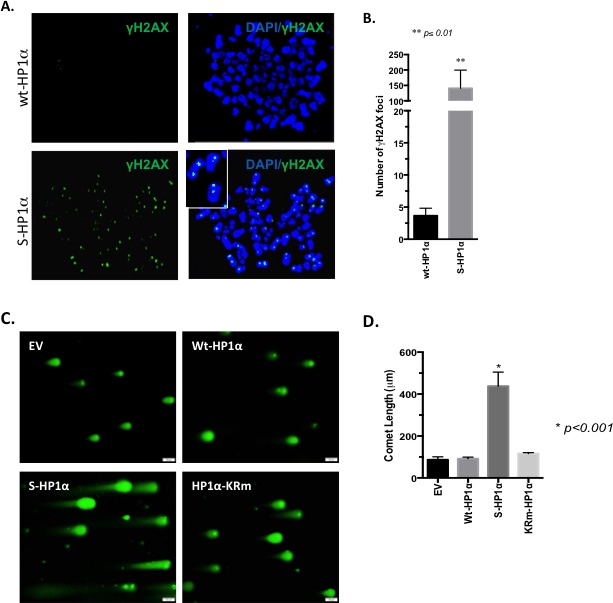
SUMOylation of HP1α promotes DNA-damage **A.**-**B.** DNA breaks persist with induction of S-HP1α. Immunofluorescence with γ-H2AX antibody indicated DNA-damage on chromosome-spread MCF7 samples. For each of the two treatment groups, three 100x magnification fields were selected at random and γ-H2AX foci present on the chromatin were counted. Student's *t*-test was used to evaluate for statistical significance; asterisk presents p-value. **C.**-**D.** SUMO-deficient HP1α does not induce DNA breaks. MCF7 cells were transfected with empty vector (EV), wild-type HP1α (wt-HP1α), SUMO3-fused HP1α (S-HP1α) or HP1α-KRm plasmid and then harvested for evaluation of DNA breaks using the comet assay. A gray scale bar at the bottom right of each image represents 50μm. **D.** The length of the comet was determined using the bar scale as a reference. Statistical significance is indicated as defined using ANOVA and Tukey's post-hoc tests.

Since the Rad51 family is a major mediator of the DDR, we postulated that S-HP1α would impair the DDR and support the accumulation of double-strand breaks (DSB) in BCa cells. MCF7 cells were assessed for γ-H2AX foci, a mark for DSB. Chromatin from cells with wt-HP1α did not exhibit incorporation of the phosphorylated H2AX variant (Figure 6A). In contrast, transfection of the S-HP1α significantly increases γ-H2AX foci on chromatin (Figure 6A and 6B). Similar results were obtained with a comet assay; specifically, elevation of S-HP1α, but not wt-HP1α, causes DNA breaks (Figure 6C and 6D). In contrast, overexpression of an HP1α mutant that is SUMO deficient (HP1α-KRm, [5]) does not damage DNA (Figure 6C and 6D). Hence, the data supports that S-HP1α reduces the ability of the cell to resolve the damaged DNA.

**Figure 7:**
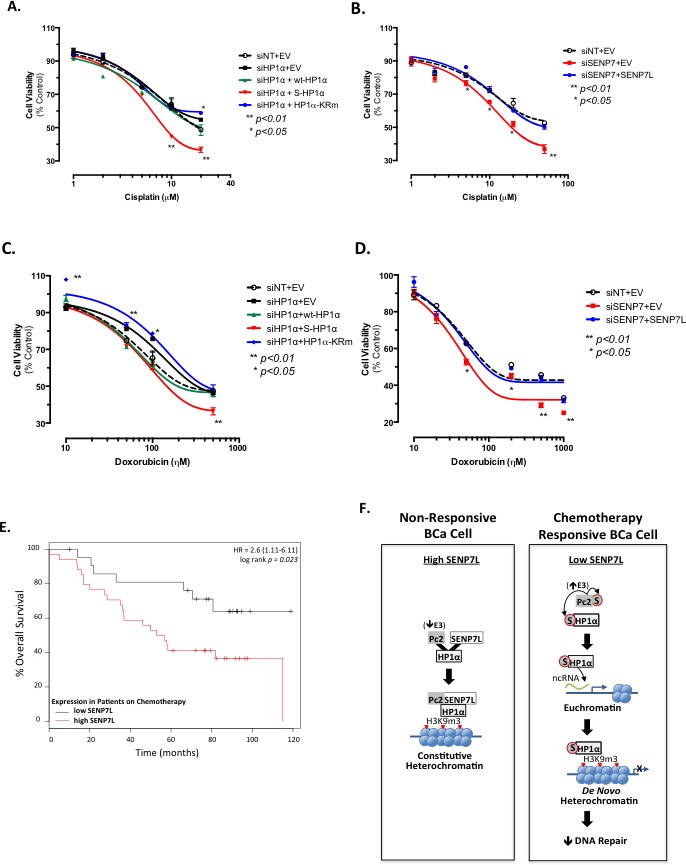
HP1α SUMOylation status and SENP7L expression predict sensitivity of BCa cells to chemotherapy **A.** HP1α SUMOylation affects cisplatin efficacy. Endogenous HP1α was knockdown in MCF7 cells with siRNA (siHP1α) and reconstituted with wt-HP1α, S-HP1α, and HP1α-KRm. Cell viability was assessed after 48h incubation with various concentrations of cisplatin. Asterisks represent statistical significance as compared to siNT+EV controls and derived by ANOVA and Dunnett's post-hoc test. **B.** Loss of SENP7L increases cisplatin efficacy. Cells were treated with either siNT or SENP7-targeting siRNA (siSENP7) concurrently with either empty vector (EV) or SENP7L prior to exposure to increasing concentrations of cisplatin. Asterisks indicate a significant difference as compared to control siNT+EV treated cells; p-values derived following ANOVA and Dunnett's post-hoc test. **C.**-**D.** Analogous transfection conditions and statistical analysis as described above was utilized to evaluate the affects of 48h doxorubicin treatment on MCF7 cell viability. S-HP1α reduces while HP1α-KRm increases the number of viable cells following doxorubicin treatment **C.**. Knockdown of SENP7 reduces cell number while reconstitution with SENP7L rescues this effect **D.**. **E.** Low SENP7L correlates with greater survival for BCa patients on chemotherapy. Kaplan-Meier plot of chemotherapy-treated BCa patients separated into low and high SENP7L gene expression levels using the KMPLOT database. HR represents the hazard ratio at 95% confidence and the p-value of *p = 0.023* indicates statistical significance. **F.** Schematic highlighting the data presented in this manuscript and explained in detail in the main text.

Rad51C and DDR regulate effectiveness of DNA-damaging chemotherapeutic agents like cisplatin [31]. Hence, we next evaluated whether the SUMOylation status of HP1α dictates sensitivity of BCa cells to cisplatin treatment. Cisplatin concentration-response assessment of MCF7 cells was performed following knockdown of endogenous HP1α and reconstitution of cells with wild-type, SUMO-fused, or SUMO-deficient HP1α (wt-HP1α, S-HP1α, and HP1α-KRm, respectively Supplemental Figure S4A and Figure 7A). Induction of S-HP1α significantly increased while HP1α-KRm decreased cisplatin efficacy (ANOVA, Dunnett's post-hoc test, *p < 0.01* and *p < 0.05* respectively, Figure 7A). Consistently, loss of SENP7L, which potentiates HP1α SUMOylation (Supplemental Figure S2A), also enhances the response of BCa cells to cisplatin treatment (*p < 0.01*, ANOVA and Dunnett's test, Figure 7B). Treatment with an anthracycline-based chemotherapeutic doxorubicin produces similar results with S-HP1α and SENP7L knockdown increasing doxorubicin efficacy (ANOVA, Dunnett's test for efficacy *p < 0.01,* Figure 7C and 7D). Using two publically available data-sets (GSE1456 and GSE3494, *n* = 56), we next evaluated the correlation between SENP7L expression and overall survival of patients on systemic chemotherapy treatment. SENP7L levels inversely correlate with overall survival for these patients; individuals on chemotherapeutics with low SENP7L mRNA exhibit significantly longer survival time than those patients with high SENP7L mRNA levels (*p = 0.023,* Figure 4F). Interestingly, these patients on chemotherapy with low SENP7L also have a greater probability of relapse-free survival (*p = 0.126*, Supplemental Figure S4B). Hence, it is likely that low SENP7L levels would indicate good response to chemotherapy.

## DISCUSSION

*In vitro* studies report the high binding affinity of PC2 for H3K9m3 over H3K27m3 peptides [8]. However, *in vivo* studies have focused primarily on PC2's recruitment to and function at H3K27m3 sites. We now demonstrate that PC2 is localized at endogenous H3K9m3 sites in BCa cells (Figure 3C and Figure 3D). Collectively the data suggests that enrichment of PC2 to H3K9m3 is dependent on 1) interaction with HP1α and 2) the SUMOylation status of PC2. First, PC2 exhibits a canonical HP1α binding motif that is required for PC2-HP1α association (Figure 1B and 1C). Second, HP1α preferentially associates with hypo-SUMOylated PC2 (Figure 2C and Supplemental Figure S1C). Third, HP1α loss reduces PC2-H3K9m3 association (Figure 3C). Fourth, SUMO3 induction enhances PC2 SUMOylation (lane 2, Figure 2B) but reduce PC2's interaction with both HP1α and H3K9m3. In BCa cells, SUMOylated PC2 is recruited to H3K27m3 (Figure 3C and Supplemental Figure S3C); similarly a previous study reports SUMOylation of PC2 increases H3K27m3 binding in mouse embryonic fibroblasts [9]. Hence while SUMOylated PC2 is enriched at H3K27m3, un-modified PC2 associates with HP1α at H3K9m3 sites.

SUMOylated PC2 at H3K27m3 functions to recruit additional PRC1 complex members, specifically Ring1b and Bmi1, to repress transcription at specific gene loci [9]. In contrast, H3K9m3 enrichment serves as a “repressive hub” to sequester un-modified PC2 and provides an alternative regulatory control of PC2's SUMO E3 ligase activity. Optimum PC2 SUMO ligase function requires two SIMs ([21] and Figure 1D). Unlike SIM1, SIM2 overlaps with a HP1α-binding site. HP1α-binding reduces PC2's SUMO ligase activity as demonstrated in Figure 1D and 1E. Exactly how PC2-SIMs regulate ligase function is unclear; it is proposed that the SIMs could either modulate association with SUMO-loaded Ubc9 or orient SUMO for transfer from Ubc9 to substrate [21]. HP1α could masks PC2-SIM2 and through steric hindrance, perturbs its full SUMO ligase activity. Alternatively, binding to HP1α could also segregate PC2 from SUMO, SUMO-bound Ubc9, and/or additional substrates. In either case, PC2-HP1α interaction would affect SUMO-PTM of multiple PC2 target proteins and regulate global levels of SUMO-conjugation in the system.

SUMOylated HP1α binds multiple euchromatin sites and exhibits a unique binding profile compared to unmodified HP1α. While HP1α localizes to constitutive heterochromatin *via* association with established H3K9m3 marks, active chromatin sites lack H3K9m3. Hence it is unlikely SUMOylated HP1α is recruited to most of the euchromatin *via* its H3K9m3 reader function. Alternatively, HP1α binds α-satellite ncRNA prior to H3K9m3 at pericentromeric heterochromatin in a SUMO-dependent manner [14]. Similarly, SUMOylated HP1α efficiently binds multiple chromatin-bound ncRNA including Rad51C ncRNA. Currently, it is unknown what drives this interaction between RNA and a SUMO-modified protein. HP1α SUMOylation could initiate a conformational change in the linker domain to facilitate RNA binding. Additionally, a previous study identifies 7 critical residues of SUMO1 that allow for binding nucleic acids; 3 of these residues are conserved in SUMO3 [32]. SUMO3 could bind RNA in an analogous manner and therefore, covalent addition of SUMO3 to HP1α could potentiate RNA binding. Extensive studies, beyond, the scope of this report, are required to clearly delineate this interaction.

HP1α SUMOylation is dependent on SENP7L. We and others established that SENP7L (and HP1α deSUMOylation) ensures the canonical function of HP1α at H3K9m3-rich heterochromatin sites [5, 7, 24]. In contrast, SUMOylated HP1α functions as a transcriptional repressor at multiple active promoter sites including DDR gene loci. Consistently, the reduced DNA repair defines DSB that persists in hyperSUMOylated HP1α conditions in BCa cells. A recent report suggests HP1α recruits SENP7L to the sites of DNA damage; interestingly, SENP7L mediates chromatin relaxation at DNA damage foci independent of HP1α deSUMOylation [6]. Collectively, the SENP7L-HP1α interaction directs resolution of damaged DNA *via* 1) epigenetic regulated gene expression and/or 2) direct chromatin remodeling. Hence, it is not surprising that SENP7L and HP1α SUMOylation define the efficacy of DNA-damaging chemotherapeutics in BCa cells. SENP7L levels predict the success rate of chemotherapeutics; specifically overall survival is increased over a 10-year period.

Collectively our data demonstrates that the SUMO dynamics of the epigenetic remodeler HP1α are tightly controlled in BCa cells and could predict chemotherapy efficacy as illustrated in Figure 7F. SUMO protease SENP7L is enriched at constitutive heterochromatin loci through its interaction with HP1α and maintains hypoSUMOylated HP1α levels in multiple cell types [5–7, 24]. The SUMO E3 ligase PC2 also associates with the heterochromatic H3K9m3 mark (Figure 3C and 3D). In BCa cells with high SENP7L levels, HP1α and PC2 are maintained in the unmodified state and readily interact. The interaction of PC2 with HP1α reduces PC2's SUMO E3 ligase activity (Figure 1D) but supports PC2's localization at H3K9m3 sites (Figure 3C and 3D). Inversely, low SENP7L levels decrease PC2-HP1α association (Figure 2C); this, in turn, enhances PC2's SUMO E3 ligase activity and SUMOylation of its target HP1α. SUMOylated HP1α is more readily bound to active chromatin sites (Figure 4). The *Rad51C* gene locus is identified as a novel binding site for HP1α (Figures 4C and 5A). SUMOylated HP1α is enriched at the *Rad51C* gene and the recruitment of SUMOylated HP1α to the *Rad51C* promoter requires chromatin-bound RNA (Figure 5A) specifically the chromatin-bound Rad51C ncRNA (Figure 5B). Recruitment of SUMOylated HP1α to these loci substantially reduces the transcription of *Rad51C* and other DNA-repair genes to potentiate DSB (Figure 5C and Figure 6). Consistently, BCa cells with elevated SUMO-modified HP1α are more sensitive to DNA-damaging chemotherapeutics agents (Figure 7). Also patients on systemic chemotherapeutics with low SENP7L levels exhibit longer disease-free survival.

Although relatively efficacious, the majority of chemotherapeutic agents exhibit adverse toxicity. Hence, predicting chemotherapy sensitivity in BCa patients serves as an illusive holy grail in treatment. SENP7L and its regulation of HP1α SUMOylation could be a good prognostic marker for chemotherapy responsiveness of BCa patients. Clearly more comprehensive studies with greater patient samples are required to validate this trend.

## MATERIALS AND METHODS

### Immunoprecipitation and western blotting of chromatin-bound proteins

Isolation of chromatin-bound (CB) proteins was performed as defined previously [5]. The sample supernatant was incubated with antibodies for HP1α and PC2 overnight and subsequently evaluated *via* SDS-PAGE.

Cells were harvested, subject to SDS-PAGE, and immunoblotted as described previously [5, 33].

### *In vitro* SUMOylation

*In vitro* SUMOylation was conducted using modifications to the protocol provided with the SUMOlink kit (Active Motif, Carlsbad, CA). Briefly, proteins were immunoprecipitated from cell lysates using the anti-Flag antibody bound to Protein A-agarose beads (Millipore, Billerica, MA). Immunoprecipitated proteins were incubated with the appropriate recombinant proteins and buffers as directed in the kit. After washes to reduce nonspecific interactions, proteins were resolved by SDS-PAGE and analyzed by immunoblotting with antibodies against HP1α and SUMO2/3.

### Chromatin immunoprecipitation (ChIP), ChIP-Seq, and RNase-ChIP

The previously established protocols were used to conduct ChIP experiments [5]. Select ChIP samples were subject to Next Generation Sequencing. For this, the MDACC Sequencing and Microarray Core Facility prepared the DNA library construction and subsequently sequenced on the Illumina platform.

The sequencing reads were aligned to hg19 reference genome. Sequencing quality and calculate the mapping statistics was assessed with SAMSTAT. Partek Genomics Suite 6.6 was used to identify the binding regions. The detected peaks are filtered according to the p-values and the FDR set to 0.05. The p-value was generated by comparing each ChIP sample to the Input-control using a one-tailed binomial test.

For RNase-ChIP experiments, appropriately treated cells were incubated at room temperature for 5 minutes with 0.5% Triton X-100 (in PBS) to initiate permeabilization. Then permeabilized cells were treated with 5-Units RNase-H at room temperature for 5 minutes. Prior to initiating the ChIP process, the cells were fixed in 1% formaldehyde at room temperature for 10 minutes. Primers used real-time PCR to identify select gene promoter regions are provided with corresponding references (Supplemental Table S3, [5, 34, 35]).

### Chromatin RNA immunoprecipitation (RIP)

Chromatin-RNA-Protein complexes were eluted and purified as directed in the RNA ChIP-it kit (Active Motif). Subsequently purified RNA was analyzed using real-time PCR and select primers for select ncRNA; list of primers and corresponding reference are included in Supplemental Table S4 [27, 36–39].

### Immunofluorescence, mitotic spread, and comet assay

Immunofluorescence of whole cells and chromosome spreads were performed as outlined in previous publications [5]. The comet assay was performed according to manufacturer's instructions (Trevigen).

### Cell viability

Cell viability was performed using the MTT assay as defined previously [40].

### Statistical analysis

GraphPad Prism Version 4.0 (GraphPad Software) was used to conduct statistical analyses with either the Student's *t-test* or ANOVA employed to determine *p* values of raw data. The Tukey's or Dunnett's post hoc test was performed to evaluate differences between groups or *versus* control with ANOVA, respectively. ChIP-Seq data was aligned by Burrows-Wheeler Aligner (BWA), a popularly used alignment tool [41]. The analysis was then performed using Partek software (Partek Inc).

## SUPPLEMENTARY MATERIAL TABLES AND FIGURES


