# Supplementary material for: SUMOylation of HP1α supports association with ncRNA to define responsiveness of breast cancer cells to chemotherapy
Source: Oncotarget. 2016 Apr 14;7(21):30336–49. doi: 10.18632/oncotarget.8733 (PMC5058684; doi:10.18632/oncotarget.8733)
Supplement: Supplementary file 1 [file oncotarget-07-30336-s001.pdf]

SUMOylation of HP1α supports association with ncRNA to define responsiveness of breast cancer cells to chemotherapy

Supplementary Material

Supplemental Table S1: Summary of Alignment Results

Sample ID	Total Reads	Mapped Reads	Mapping Rate %
Input-wt-HP1α	17.0M	14.7M	86%
Input-S-HP1a	19.8M	17.7M	89%
wt-HP1a	11.3M	10.3M	91%
S-HP1a	31.3M	28.5M	91%

Supplemental Table S2: Number of Peaks Detected Independent of Input

Sample ID	FDR 0.05	FDR 0.05 & No. Overlap with Input
wt-HP1a	823	632
S-HP1a	1155	1012

Supplemental Table S3: Primer List for RNase-ChIP.

Chromatin Loci	Primers	Reference
DHFR promoter	Forward: 5’-TTCTGCTGTAACGAGCGGGCTCGGA-3’<br>Reverse: 5’-CTACAAGTTAGAGAAACAGCGTTACTCGAA-3’	5
TS promoter	Forward: 5’-TGGCGCACGCTCTCTAGAGC-3’<br>Reverse: 5’-GACGGAGGCAGGCCAAGTG-3’	5
c-Myc promoter	Forward: 5’-GGCTTCTCAGAGGCTTGGCGGG-3’<br>Reverse: 5’-TCCAGCGTCTAAGCAGCTGCAA-3’	30
Rad51C promoter	Forward: 5’-CGCTTGCACTGATCACCAGA-3’<br>Reverse: 5’-GTTGCCTGCCTCTGCATTTG-3	-
BRIP1 promoter	Forward: 5’-CTGTCATATACCTACTGCCCAG-3’<br>Reverse: 5’-GAGGTGTGATGATGGGATGGCA-3’	-
PPM1D promoter	Forward: 5’-CAGTTGGGGTTAAGCCATGTTG-3’<br>Reverse: 5’-GGGGTGGCTCACGTCTATAATC-3’	-
Vimentin promoter	Forward: 5’-CCGCAGCCCCGAGACCGCCGCGCA-3’<br>Reverse: 5’-GTCCCGTTACTTCAGCGCTGGGCT-3’	5
c-fos promoter	Forward: 5’-TGTTGGCGGCAGCCCGCGAGCAGTTC-3’<br>Reverse: 5’-GGCGCGTGTCCTAATCTCGTGAGCAT-3’	5
Telomere	Forward: 5’-GGTTTTTGAGGGTGAGGGTGAGGGTGAGGGTGAGGGT-3’<br>Reverse: 5’-TCCCGACTATCCCTATCCCTATCCCTATCCCTATCCCTA-3’	29
α-satellite	SimpleChIP® Human α Satellite Repeat Primers #4486	Cell Signaling Technology

Supplemental Table S4: Primer List for Chromatin RIP.

RNA	Primers	Reference
DHFR ncRNA	Forward: 5’-TTGCCCTGCCATGTCTCG-3’<br>Reverse: 5’-ACCTGGTCGGCTGCACCT-3’	24
Rad51C ncRNA	Forward: 5’-TGTTGGAAAAACACAATTATGGTAA-3’<br>Reverse: 5’-TCTTATTTGGTTTCCTGACGA-3’	-
HOTAIR	Forward: 5’- CAGTGGAATGGAACGGATTT -3’<br>Reverse: 5’- TCAGACTCTTTGGGGCCTTA -3’	31
Xist	Forward: 5’-CTTGAAGACCTGGGGAAATCCC-3’<br>Reverse: 5’-TGTCAATCTAAAGGTAACCGGC-3’	32
TUG	Forward: 5’-AGTGAATTATGTCCTGTGCCT-3’<br>Reverse: 5’-GATGGGTGAATGCCTCCTG-3’	33
chromosome 2p TERRA	Forward: 5’-TAAGCCGAAGCCTAACTCGTGTC-3’<br>Reverse: 5’-GTAAAGGCGAAGCAGCATTCTCC-3’	34

Supplemental Table S5: Primer List for Real-time PCR.

RNA	Primers	Reference
DHFR mRNA	Forward: 5’-TAAACTGCATCGTCGCTGTGT-3’<br>Reverse: 5’-AGGTTGTGGTCATTCTCTGGAAA-3’	5
Rad51C mRNA	Forward: 5’-CTGCATTAGGGGAAAGTTGG-3’<br>Reverse: 5’-GGGTGCTCAAGGAACCTTCT-3’	-
PPM1E mRNA	Forward: 5’-TCAATCCATGCCATCAAAAA-3’<br>Reverse: 5’-GAACATCTCCTGGCGGACTA-3’	-
PPM1D mRNA	Forward: 5’-AGCACTTGTGGGGTTTCATC-3’<br>Reverse: 5’-TACATCTTCATGCCCCGAAT-3’	-
BRIP1 mRNA	Forward: 5’-CCCCGTTTCAAAACAGAGAA-3’<br>Reverse: 5’-CCAGGGCTTCTTCAGAACAG-3’	-

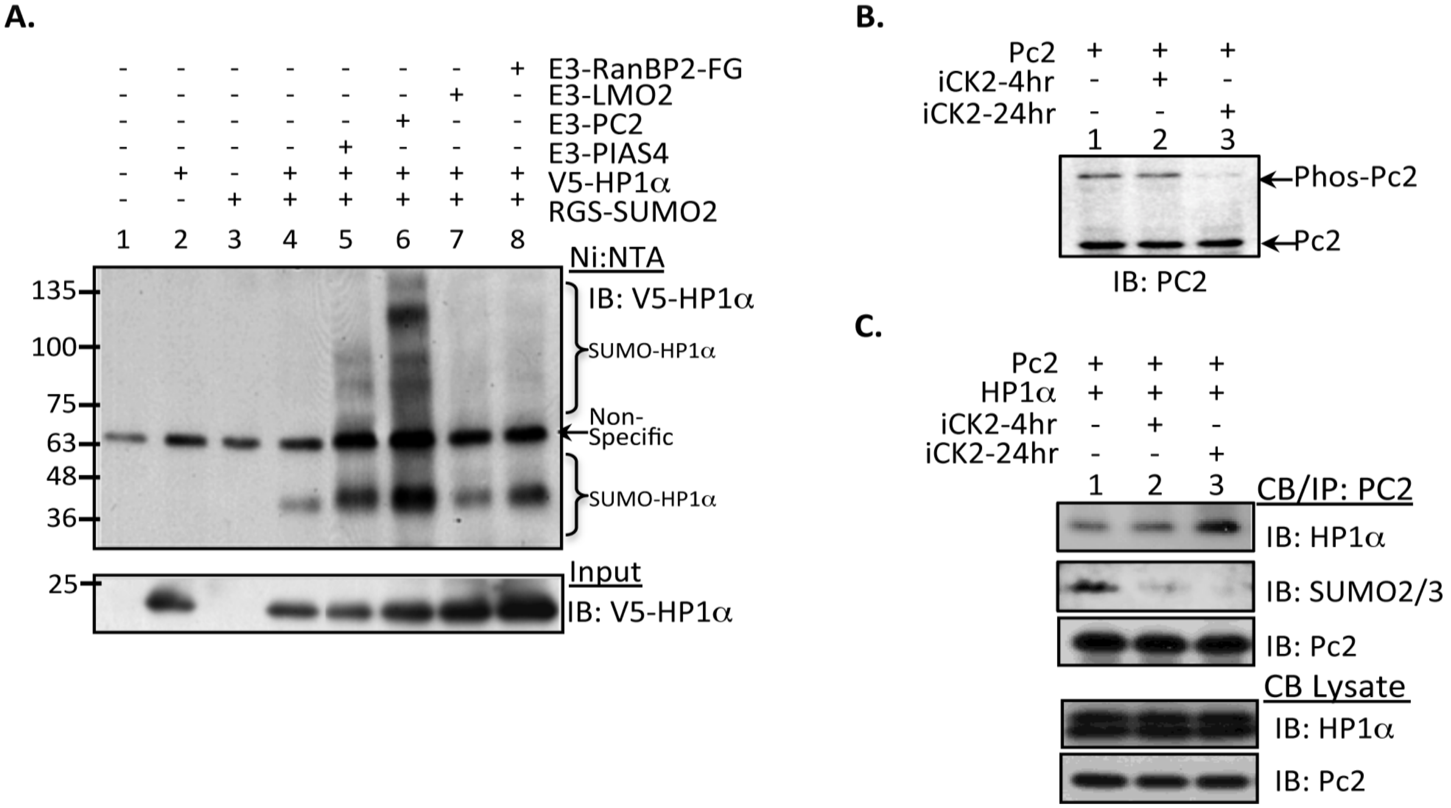

Supplemental Figure S1: *PC2's SUMO E3 ligase activity and HP1α knockdown efficiency.* (**A**) PC2 exhibits SUMO E3 ligase activity for HP1α. HEK293 cells were transfected with equivalent amounts of the indicated plasmids in the presence of select SUMO E3 ligases. SUMOylated proteins were purified with a nickel-agarose resin and subject SDS-PAGE. Immunoblots for the V5-tag of HP1α is presented from Ni:NTA and Input samples. (**B-C**) MCF7 cells were treated with the CK2 inhibitor for the indicated time points prior to harvesting. (**B**) Samples were subject to Phos-tag SDS-PAGE to identify phosphorylated PC2, which is reduced with 24hr CK2 inhibitor treatment. (**C**) PC2 was immunoprecipitated from chromatin fractions and subsequently assessed for SUMO-PTM and HP1α-interaction. Treatment with CK2 inhibitor reduces PC2 auto-SUMOylation (correlating with reduced SUMO-E3 activity) but increases PC2-HP1α interaction at the chromatin.

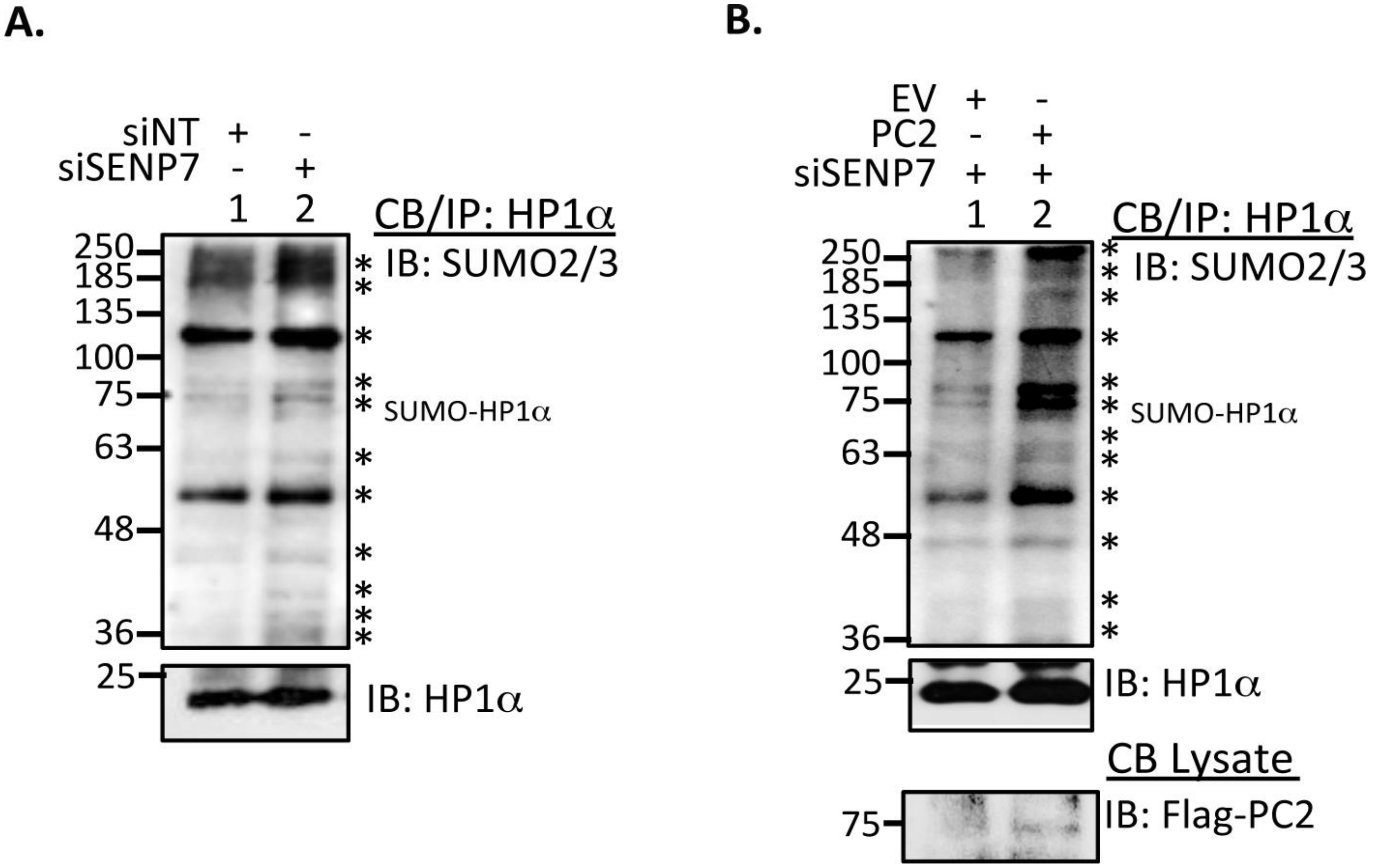

Supplemental Figure S2: Endogenous HP1α is hyperSUMOylated with knockdown of SENP7 and induction of PC2. (**A**) MCF7 cells were treated with non-targeting or SENP7-targeting siRNA for 24hr and endogenous HP1α was immunoprecipitated from chromatin fractions. SUMO-PTM HP1α that increases with siSENP7 treatment is highlighted with asterisks. (**B**) siSENP7-treated MCF7 cells were concurrently transfected with empty vector (EV) or Flag-tagged PC2. Endogenous chromatin-bound HP1α was isolated and evaluated for SUMO-PTM. The increase SUMOylated HP1α observed with PC2 induction is marked with asterisks.

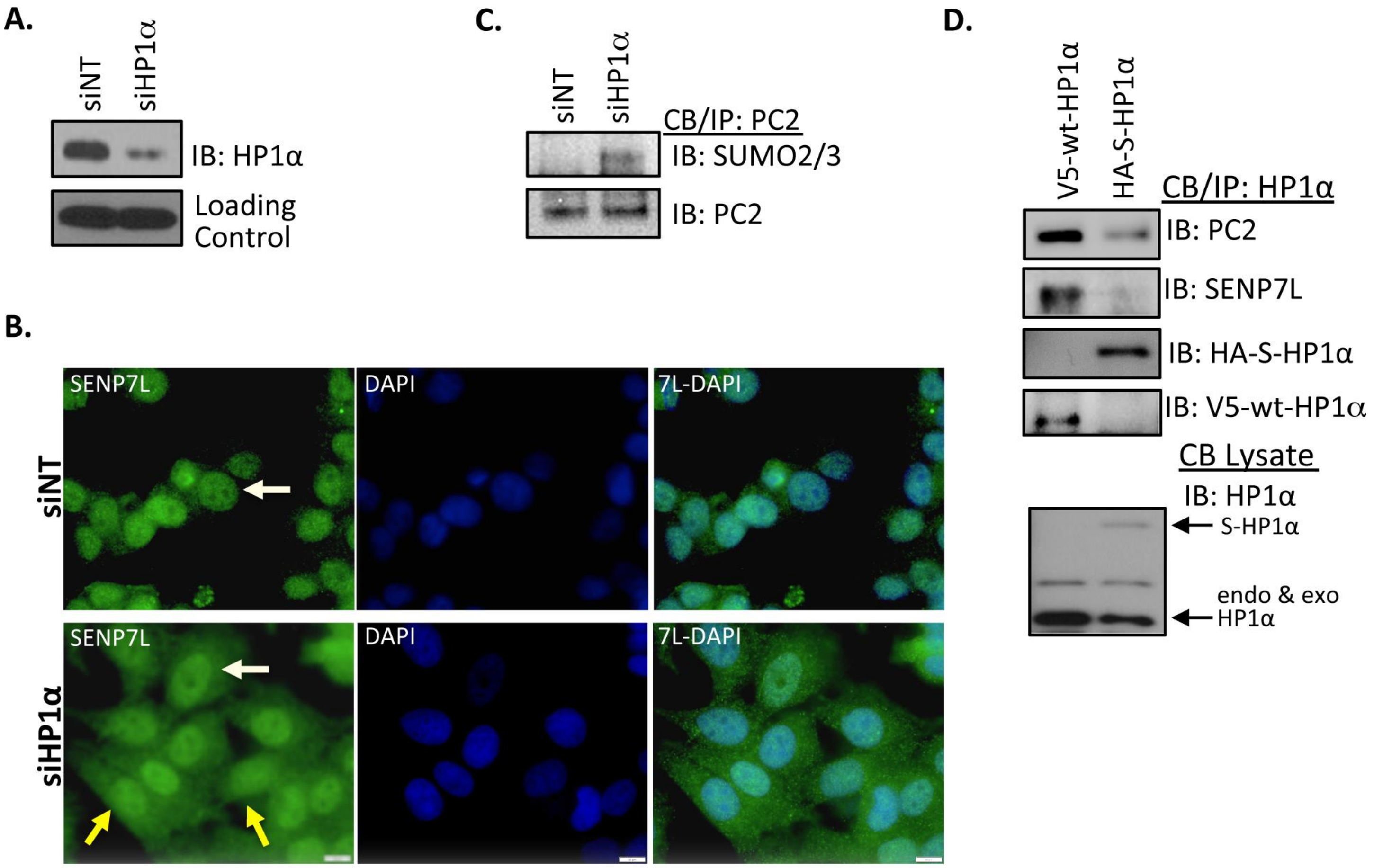

Supplemental Figure S3: MCF7 cells were treated with either non-targeting (siNT) or HP1α-targeting siRNA (siHP1α). Cells were utilized after 48h in normal growth conditions. (**A**) HP1α protein loss with targeted siRNA treatment. (**B**) SENP7L redistribution with siHP1α. Multiple 20x magnification images were assessed for SENP7L localization; specifically, percent of cells with 1) greater nuclear to cytosolic (white arrows), 2) equal nuclear and cytosolic (yellow arrows), and 3) greater cytosolic to nuclear SENP7L was evaluated. (**C**) HP1α loss increases PC2 SUMOylation. Chromatin-bound PC2 was immunoprecipitated and evaluated for SUMO2/3 modification via western blot with the indicated antibodies. (**D**) Neither PC2 nor SENP7L efficiently bind S-HP1α. Immunoprecipitated wt- and S-HP1α were evaluated for interaction with PC2 and SENP7L using Western blot analysis. All blots are representative of 2-3 independent experiments.

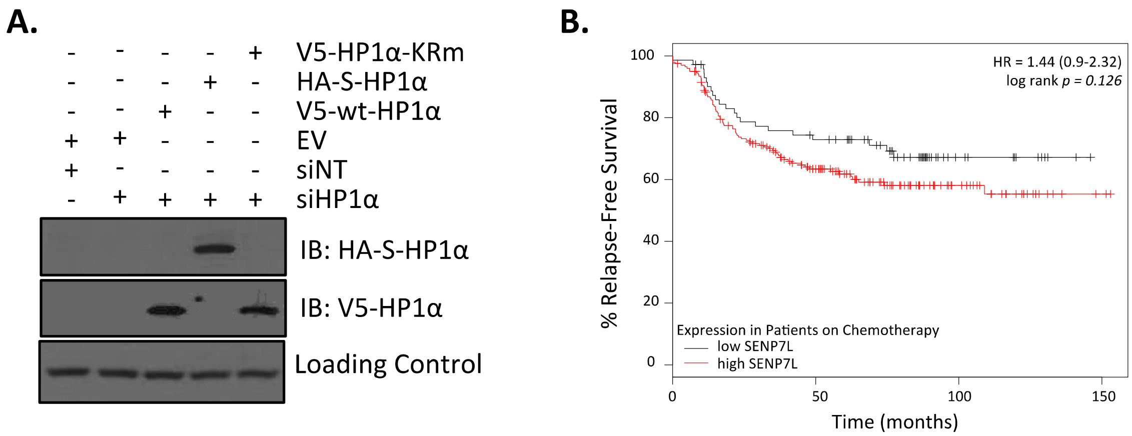

Supplemental Figure S4: HP1α SUMOylation and consistently SENP7L loss increases probability of recurrence-free disease. (**A**) MCF7 cells were treated with the indicated siRNA (siNT, siHP1α, or siSENP7) and concurrently transfected with either empty vector (EV) or the indicated plasmid construct. After 48hr, cells were evaluated for plasmid expression via SDS-PAGE. (**B**) BCa patients on chemotherapy with low SENP7L exhibit a lower risk of disease recurrence or relapse. Kaplan-Meier plot of chemotherapy-treated BCa patients separated into low and high SENP7L gene expression levels indicates a statistical trend towards significance with a p-value of $p=0.126$.
